# Pathogenic or Therapeutic Extracellular Vesicles in Rheumatic Diseases: Role of Mesenchymal Stem Cell-Derived Vesicles

**DOI:** 10.3390/ijms18040889

**Published:** 2017-04-22

**Authors:** Stella Cosenza, Maxime Ruiz, Marie Maumus, Christian Jorgensen, Danièle Noël

**Affiliations:** 1 Institute of Regenerative Medicine and Biotherapies, INSERM, University of Montpellier, 34090 Montpellier, France; stella.cosenza@inserm.fr (S.C.); maxime.ruiz@inserm.fr (M.R.); marie.maumus@inserm.fr (M.M.); christian.jorgensen@inserm.fr (C.J.); 2 Clinical Immunology and Osteoarticular Diseases Therapeutic Unit, Hôpital Lapeyronie, 34090 Montpellier, France

**Keywords:** osteoarthritis, rheumatoid arthritis, extracellular vesicle, exosome, microparticle, mesenchymal stem cell, cell therapy

## Abstract

Extracellular vesicles (EVs) are important mediators of cell-to-cell communication pathways via the transport of proteins, mRNA, miRNA and lipids. There are three main types of EVs, exosomes, microparticles and apoptotic bodies, which are classified according to their size and biogenesis. EVs are secreted by all cell types and their function reproduces that of the parental cell. They are involved in many biological processes that regulate tissue homeostasis and physiopathology of diseases. In rheumatic diseases, namely osteoarthritis (OA) and rheumatoid arthritis (RA), EVs have been isolated from synovial fluid and shown to play pathogenic roles contributing to progression of both diseases. By contrast, EVs may have therapeutic effect via the delivery of molecules that may stop disease evolution. In particular, EVs derived from mesenchymal stem cells (MSCs) reproduce the main functions of the parental cells and therefore represent the ideal type of EVs for modulating the course of either disease. The aim of this review is to discuss the role of EVs in OA and RA focusing on their potential pathogenic effect and possible therapeutic options. Special attention is given to MSCs and MSC-derived EVs for modulating OA and RA progression with the perspective of developing innovative therapeutic strategies.

## 1. Introduction on Extracellular Vesicles

Extracellular vesicles (EVs) are heterogeneous small vesicles surrounded by a phospholipid bilayer. They are secreted by virtually all cell types and are found in various biological fluids (blood, urina, saliva, cerebrospinal fluid, breastmilk and others). Recently, EVs are described as a main mechanism involved in cell-to-cell communication. Upon release in the extracellular space, they can reach the circulation and act at distant sites where they discharge their cargo into recipient cells and reproduce the effect of the parental cells. 

There exist several types of EVs but vesicles commonly described are exosomes, microparticles (or microvesicles) and apoptotic bodies. These different types of EVs can be classified according to their size, their composition and also their origin [1]. Exosomes are small vesicles with a size between 80 to 150 nm. They are constitutively secreted by all cell types and derive from the endosomal compartment. In the late endosome, they arise from endosomal membrane invagination that forms intraluminal vesicles inside of the endosomal compartment. This structure is called multivesicular body (MVB). During invagination of the membrane, diverse proteins, lipids and nucleic acids are selectively encapsulated into intraluminal vesicles. Then, the MVB fuses with the plasma membrane and liberates exosomes directly in the extracellular space. Because of this endosomal origin, exosomes are characterized by the expression of endosomal markers: tetraspanin proteins (CD9, CD63, and CD81) but also proteins (such as TSG101 and ALIX) from the endosomal sorting complex required for transport (ESCRT). 

Microparticles (MP), also called microvesicles, are vesicles induced after cell stimulation or a stress such as apoptosis or hypoxia. They shed directly from the plasma membrane after loss of asymmetric phospholipid distribution and cytoskeleton reorganization. They have a size between 150 and 600 nm in diameter and express membrane markers from the parental cells. Similar to exosomes, MPs also contain proteins, lipids and nucleic acids. Apoptotic bodies (AB) are the third main type of EVs. They are more than 1000 nm in diameter and are induced during the late stage of apoptosis as blebs of dead cells. We will only focus on exosomes and microvesicles in the present review.

EVs can interact with recipient cells via different mechanisms. EVs can fuse with the plasma membrane of target cells or be internalized by endocytosis or can interact with cell surface receptors and induce intracellular signaling pathways. When internalized, EVs can release proteins, lipids and also nucleic acids such as miRNA and mRNA that are functionally active inside cells. EVs exert several functions depending on the cell they originate. Indeed in a pathological context, EVs may have a pathogenic effect and promote disease progression but, conversely, EVs may be protective and prevent the development of the disease.

## 2. Extracellular Vesicles in Rheumatic Diseases

### 2.1. Pathological Role of Extracellular Vesicles in Osteoarthritis

Osteoarthritis (OA) is a disease characterized by articular cartilage degradation, alteration of bone structure, synovial inflammation and pain. All types of cells in the joint secrete EVs that could participate in the progression of the disease, by increasing inflammation and/or acting as pathological signal. As early as 1969, EVs containing hydroxyapatite crystals were identified in cartilage [2]. EVs from OA articular cartilage had a size between 50 to 250 nm and were mainly detected in the tidemark area, associated with increased alkaline phosphatase activity [3]. They not only contained pathological calcium crystals but also a decreased content in proteoglycans and modified amounts of various proteins. Since then, a recent study showed that EVs derived from interleukin (IL)-1β stimulated chondrocytes increased the production of matrix metalloproteinase (MMP)-13 by synovial-like fibroblasts [4]. Moreover, production of IL-1β, tumor necrosis factor (TNF)-α and cyclooxygenase (COX)-2 by synoviocytes was markedly enhanced indicating a role of EVs in the inflammatory process during OA.

The other main source of EVs in the joint is the synovial lining cells comprising synovial fibroblasts and macrophages. Only one publication reports the role of EVs secreted by synovial fibroblasts in OA. Kato et al. showed that exosomes from IL-1β stimulated synovial fibroblasts significantly up-regulated MMP-13 and a disintegrin and metalloproteinase with thrombospondin motifs (ADAMTS)-5 expression in articular chondrocytes and down-regulated type II collagen (COL2) and aggrecan (ACAN) compared with untreated synoviocytes [5]. In addition, IL-1β stimulated fibroblasts-derived exosomes contained higher amounts of IL-6, MMP-3 and vascular endothelial growth factor (VEGF). These exosomes were also shown to induce angiogenesis in human umbilical vein endothelial cells (HUVECs) with higher migration and tube formation activity.

Indeed, synovial fluid (SF) from OA patients contains EVs released both by synoviocytes and chondrocytes. However neither the concentration nor the size of EVs differed in OA and non-OA individuals [4]. EVs from OA SF were readily endocytosed by chondrocytes and analysis of EV cargo indicated that miR-200c was increased in OA SF-derived EVs. Interestingly, miR-200c was shown to enhance type II collagen expression and to mitigate IL-6 dependent inflammation. MiR-200c-containing EVs might therefore play a role in maintaining cartilage homeostasis by reducing inflammation and increasing anabolic activity in chondrocytes. A better understanding of EV mechanisms would contribute to decipher how EVs participate to joint homeostasis and OA pathology.

### 2.2. Therapeutic Role of Extracellular Vesicles in Osteoarthritis 

To date, there are only two reports on therapeutic approaches using EVs to slow down OA. Both rely on the use of mesenchymal stem cell (MSC)-derived exosomes and are discussed later in Section 4.3.

### 2.3. Pathological Role of Extracellular Vesicles in Rheumatoid Arthritis 

Rheumatoid arthritis (RA) is a chronic autoimmune disease characterized by synovial inflammation that leads to progressive destruction of articular cartilage. In RA, SF contains a large amount of EVs including MPs and exosomes, whose number is directly correlated with disease progression. MPs from SF originated from monocytes, granulocytes and to a smaller extent from lymphocytes (few originated from platelets and erythrocytes). MPs support thrombin generation likely contributing to local hypercoagulation in inflamed joints of RA patients [6,7]. Presence of citrullinated proteins, which are known to be autoantigens in RA, was detected in SF-derived exosomes in association with IgM and IgG, suggesting they may contribute to RA induction [8]. In addition, MPs in SF from patients with RA were shown to be potent inducers of B lymphocyte survival and release of a number of mediators such as B cell-activating factor (BAFF) or thymic stromal lymphopoietin (TSLP) by RA fibroblast-like synoviocytes [9]. 

MPs derived from platelets are found in low proportion in SF, but are highly detected in plasma from RA patients. However, platelet-derived MPs were in comparable number in plasma from RA patients or healthy controls [6]. Another report found opposite results with higher counts of platelet-derived MPs in RA plasma, which were directly associated with disease activity [10]. Different studies suspected a role of platelet cells in RA progression. Proof of pathophysiological importance of platelets was demonstrated in the K/BxN serum transfer model of inflammatory arthritis where marked reduction of arthritis severity was observed when platelets were depleted using a platelet-depleting antibody regimen [11]. In this report, platelet-derived MPs, as evaluated by expression of the platelet specific marker CD41, were found in RA SF (rheumatoid arthritis synovial fluid) but were undetectable in OA SF. Platelet-derived MPs were shown to induce release of cytokines, including IL-6 and IL-8, by fibroblast-like synoviocytes. Synovial activation occurred through the expression of MP membrane-associated IL-1α and IL-1β activity. This study clearly demonstrated the importance of platelets and platelet-derived MPs in RA by contributing to joint inflammation. More recently, platelet-derived MPs were detected inside neutrophils isolated from joints of RA patients and in a murine model of inflammatory arthritis [12]. MPs internalization was promoted via 12(S)-hydroxyeicosatetranoic acid (12(S)-HETE), a lipid mediator generated from membrane arachidonic acid by concerted activity of 12-lipoxygenase (12-LO) present in MPs and secreted phospholipase A2 IIA present in inflammatory fluids, and this mechanism contributed to inflammation. 

Among EVs-producing cells in SF, monocytes and granulocytes produced MPs that were shown to modulate the cytokine secretory profile of synovial fibroblasts through increased release of monocyte chemoattractant protein (MCP)-1, IL-6, IL-8, RANTES (or Chemokine (C–C motif) ligand 5, CCL5), intercellular adhesion molecule (ICAM)-1 and vascular endothelial growth factor (VEGF); all contributing to inflammation [7]. In addition, T lymphocyte- and monocyte-derived MPs were demonstrated to induce secretion of MMP-1, -3, -9, and -13 and up-regulate IL-6, IL-8, MCP-1, and MCP-2 by fibroblast like-synoviocytes [13]. Moreover, monocyte-derived MPs from RA patients up-regulated the production of prostaglandin E2 (PGE2) and microsomal prostaglandin E synthase 1 (mPGES-1) in synovial fibroblasts [14]. MPs were able to activate c-Jun N-terminal kinases (JNK), activator protein (AP)-1, p38 and nuclear factor-κB (NF-κB) signaling pathways in synovial fibroblasts and transport arachidonic acid to fibroblasts for its conversion to PGE2.

Synovial fibroblasts-derived EVs have also been investigated in RA and shown to induce inflammatory changes in chondrocytes. Exosomes produced by synovial fibroblasts from RA patients contained a membrane bound form of tumor necrosis factor (TNF)-α that was absent on exosomes from OA patients [15]. This membrane form of TNF-α stimulated collagenase-1 expression and NF-KB pathway in synovial fibroblasts, rendering them resistant to apoptosis. Interestingly, MPs derived from RA synovial fibroblasts contained high levels of ADAMTS-5 that may promote aggrecan destruction and contribute to the invading capacity of synovial fibroblasts through aggrecan-rich extracellular matrices [16]. Similarly, hexosaminidase D activities (*N*-acetyl-β-d-glucosaminidase and *N*-acetyl-β-d-galactosaminidase) were also detected in synovial fibroblasts-derived EVs from RA patients [17]. These glycosaminoglycans degrading glycosidases may contribute to cartilage degradation in joint diseases in collaboration with proteases [18]. These overall observations demonstrated presence of pathogenic EVs in SF from RA patients that can contribute to disease propagation/progression. More investigation into the role of EVs in RA is needed to determine the interest of using EVs as disease markers or therapeutic targets for RA. 

### 2.4. Therapeutic Role of Extracellular Vesicles in Rheumatoid Arthritis 

EVs from different cell sources have been described to exert therapeutic effects in murine models of RA. First studies were done using dendritic cells (DC)-derived exosomes in 2005 [19]. In this study using the collagen-induced arthritis (CIA) murine model, IL-10-treated DC-derived exosomes were able to suppress the onset of arthritis and reduce the severity of established arthritis. In a similar approach, the same group showed that exosomes derived from Fas ligand (FasL)-expressing DCs exerted an anti-inflammatory effect in established CIA in an antigen specific and MHC class II-dependent manner [20]. They also produced exosomes derived from DCs expressing IL-4 and again demonstrated effectiveness to treat established CIA through the modulation of antigen-presenting cells and T cell activity based on a MHC class II and FasL/Fas-dependent mechanism [21]. Finally, they used exosomes derived from DCs over-expressing the immunosuppressive enzyme indoleamine 2,3-dioxygenase (IDO) and showed an anti-inflammatory effect of the treatment in CIA [22]. 

The effect of artificial vesicles, namely synovium-specific targeting liposomes loaded with specific cargo glucocorticoids, has been investigated. In vivo delivered immunosuppressive liposomes specifically targeted fibroblast synoviocytes and a dramatic reduction in inflammation was reported in a rat model of adjuvant-induced arthritis [23].

More recently, another group demonstrated that neutrophil-derived EVs were highly recovered from RA SF as compared to that of healthy controls [24]. These EVs were shown to express large amounts of the anti-inflammatory protein annexin A1 (AnxA1). In vitro, they demonstrated that AnxA1^+^ EVs activated expression of anabolic genes in chondrocytes. In vivo in inflammatory arthritis, intra-articularly injected EVs localized within the cartilage matrix and protected cartilage from loss of sulfated glycosaminoglycans (sGAGs). Cartilage protection was induced upon AnxA1-expressing EVs interaction with its receptor *N*-formyl peptide receptor 2 (FPR2) and tumor growth factor (TGF)-β pathway activation. Indeed, the interest of using EVs isolated from neutrophils or DCs to reduce inflammatory arthritis has been demonstrated. However, even though therapeutic efficacy of mesenchymal stem cells (MSC) has been demonstrated in arthritis [25], no reports exist on the role of MSC-derived EVs in inflammatory arthritis. 

## 3. Mesenchymal Stem Cells and Rheumatic Diseases 

### 3.1. Characteristics of Mesenchymal Stem Cells 

Multipotent mesenchymal stem or stromal cells (MSCs) are adult stem cells isolated from a variety of tissues including bone marrow, adipose tissue, dental pulp, umbilical cord (UC) and others, but the two main sources for therapeutic utilization are bone marrow (BM-MSC) and adipose tissue (ASC) [26]. MSCs are defined by the International Society for Cellular Therapy (ISCT) by three criteria: they are adherent to plastic; they express CD73, CD90, and CD105 and are negative for CD14, CD11b, CD34, CD45, CD79a, and human leukocyte antigen (HLA)-DR; and they can differentiate into osteoblasts, adipocytes and chondrocytes [27]. In addition to their differentiation potential, MSCs also exert anti-inflammatory, proliferative, anti-apoptotic, anti-fibrotic, angiogenic functions that are mediated via the secretion of a large variety of trophic factors, including growth factors, cytokines, chemokines [28]. 

### 3.2. Differentiation and Trophic Potential of Mesenchymal Stem Cells 

Chondrogenic differentiation potential and paracrine functions of MSCs make these cells relevant for cell therapy and tissue engineering approaches for cartilage repair in osteochondral defects and osteoarticular diseases, including RA and OA. In vitro, MSCs differentiate into chondrocytes under specific culture conditions, and generate differentiated cells able to secrete a cartilaginous extracellular matrix (ECM) with properties close to naïve hyaline articular cartilage, even though some hypertrophic features are also found. To obtain more functional mature chondrocytes, several approaches have been developed to combine MSCs with biomimetic scaffolds and growth factors in order to support chondrogenic differentiation and generate fully functional hyaline articular cartilage in pre-clinical animal models [29]. 

Trophic functions of MSCs have been extensively investigated. MSCs were demonstrated to promote cell survival and protect against apoptosis induced by trauma, oxidative stress or chemical injury by the secretion of insulin growth factor (IGF)-1, TGF-β1, stanniocalcin-1 and IL-6 [30]. The anti-fibrotic effect of MSCs has been largely documented and shown to be mediated in part by production of hepatocyte growth factor (HGF), basic fibroblast growth factor (bFGF) and adrenomedullin [31]. HGF acts on the balance between anti-fibrotic MMPs and tissue inhibitor of MMPs (TIMPs), which play important roles in fibrosis resolution. MSCs were described for their resistance to oxidative stress through secretion of anti-oxidative molecules such as heme oxygenase (HO)-1 and erythropoietin (EPO) that modulate production of anti-oxidant molecules, including superoxide dismutase (SOD) or glutathione peroxidase (GSH-Px), in injured tissues [32]. The most described function of MSCs is likely their anti-inflammatory role. After exposure to an inflammatory environment, MSCs acquire immunosuppressive properties and modulate cells from both innate and adaptive immune system. MSCs are activated upon exposure to interferon (IFN)-γ in presence of TNF-α, IL-1β or IL-1α [33]. These cells inhibit proliferation and differentiation of CD4^+^ and CD8^+^ T lymphocytes and induce CD4^+^CD25^+^Foxp3^+^ and IL-10-expressing T regulatory cells. They reduce activation, proliferation and differentiation of B lymphocytes into antibody-secreting plasma cells [34]. They inhibit activation and maturation of dendritic cells (DC) by impairing the toll-like receptor (TLR) activation and induce a tolerogenic profile with production of IL-10 [35]. MSCs induce a switch from pro-inflammatory M1-like to M2-like macrophage subsets and inhibit natural killer (NK) cell activation [36,37]. These immunosuppressive functions are mediated by contact-dependent mechanisms but primarily through the release of regulatory mediators such as kynurenine produced by indoleamine 2-3-dioxygenase (IDO), nitric oxide (NO), IL-6, PGE2, TNF-inducible gene (TSG)-6, HLA-G5, IL1 receptor antagonist (IL1RA) [38]. Indeed, thanks to the plethora of mediators they secrete and paracrine functions they exert, MSCs are obvious candidates for therapeutic application in rheumatic diseases, in particular for patients with OA and RA.

### 3.3. Therapeutic Efficacy of Mesenchymal Stem Cells in Preclinical Models of Rheumatic Diseases

Therapeutic potential of intra-articular injection of a suspension of MSCs has been intensively investigated in experimental models of osteo-articular diseases. In OA, the first study reported the effect of a single and local injection of BM-MSCs after complete excision of the medial meniscus and resection of the anterior cruciate ligament in a caprine model [39]. Direct evidence of regeneration of the medial meniscus was obtained and injected cells were detected in the newly formed tissue. Treated animals exhibited reduction of articular cartilage degeneration, osteophytic remodeling and subchondral sclerosis [39]. This study demonstrated the proof-of-concept that single suspension of MSCs without any scaffold could prevent degradation of articular cartilage in OA. In a rat model of massive meniscal defects, Horie et al. showed that intra-articular injection of synovial-MSCs promoted meniscal regeneration [40]. Later on in the same model, the authors showed that intra-articular injection of human MSCs contributed to meniscal regeneration via Hedgehog signaling, which was essential in enhancing the expression of type II collagen [41]. In the collagenase-induced murine model of OA (CIOA), a single intra-articular injection of ASCs at early-stage inhibited synovial thickening and cartilage destruction likely by inhibiting synovial macrophage activation [42]. Since then, several studies confirmed benefit of local injection of MSCs in different models, including rabbit, dog and goats [43,44,45]. Interestingly, addition of platelet-rich-plasma (PRP) enhanced the therapeutic efficacy of co-injected MSCs [46,47].

In RA, a first study described that a single injection of primary murine MSCs prevented the onset of CIA, which was associated with a decrease in serum pro-inflammatory cytokines and an increase in Treg cells [48]. Immunomodulatory role of murine BM-MSCs was reported to be dependent on the expression of IL-6-dependent PGE2, IL1-RA and glucocorticoid-induced leucine zipper (GILZ) while inducible nitric oxide synthase (iNOS) expression was dispensable [49,50,51]. However, some studies failed to demonstrate any improvement with MSC treatment, probably related to mouse strain genetic background, or use of immortalized murine MSCs [52,53,54]. Subsequently, confirmation of therapeutic benefit of MSCs has been reported in many reports using MSCs from different sources (umbilical cord, adipose tissue, gingiva, BM) or origin (human, rat, mouse) [55,56,57,58,59]. Nowadays, there is no doubt that MSCs can exert an anti-inflammatory role in inflammatory arthritis.

### 3.4. Therapeutic Efficacy of Mesenchymal Stem Cells in Clinics

Based on the large amount of published results in a variety of autoimmune and degenerative diseases, MSCs might also become novel cell therapy options for the treatment of rheumatic diseases. In RA, four MSC-based clinical trials were registered on the National Institute of Health (NIH) clinical trial database (http://clinicaltrials.gov/). Only one of them is completed while two are not recruiting. No published data are available at the moment. In OA patients, most of past and current clinical trials have evaluated safety of MSC injection in phase I/II trials with some encouraging results. In February 2017, fifty-three MSC-based clinical trials were registered for OA treatment on the NIH clinical trial database. Most of them were in phase I or II. A publication from our group has recently reviewed the literature on completed clinical trials in OA [45]. More recent publications in 2016 reported the results of the ADIPOA phase I trial evaluating the safety of an escalating dose of autologous ASCs and a phase I/II trial comparing two doses of BM-MSCs to active control in patients with knee OA [60,61]. Both studies reported safety and tolerability of single MSC injection with improvement of Western Ontario & McMaster universities Arthritis index (WOMAC) and Visual Analog Scale (VAS) scores and some evidence of functional improvement. However, efficacy in double blind randomized phase IIB/III trials is still lacking.

## 4. Extracellular Vesicles Derived from Mesenchymal Stem Cells: Interest in Rheumatic Diseases 

In recent years, the emergence of EVs as a new way to cell-cell communication has gained much attention. Similar to other cell types, MSCs secrete EVs that can mirror the effect of parental cells by interacting with other cells through transfer of information. MSC-EVs have been shown to exert immunosuppressive, pro-angiogenic, anti-apoptotic and anti-fibrotic effects supporting their use in various pathologies, including rheumatic diseases. In the following sections, EVs will refer to all types of vesicles, without specifying whether exosomes, microparticles or total EVs were used.

### 4.1. Immunosuppressive Function of Extracellular Vesicles Derived from Mesenchymal Stem Cells

As discussed before, immunosuppressive functions of MSCs are of high interest in the case of inflammatory diseases such as RA. While therapeutic effect of MSC-EVs in preclinical models of RA is not yet documented, some studies have investigated the immunomodulatory effects of MSC-EVs in vitro. First report on the immunosuppressive role of MSC-EVs showed reduction of T lymphocyte proliferation, increased secretion of IL-10 and TGF-β1 and increased number of apoptotic lymphocytes in splenocytes isolated from experimental autoimmune encephalomyelitis (EAE) [62]. MSC-EVs also promoted generation of CD4^+^CD25^+^Foxp3^+^ regulatory T cells. These MSC-EVs expressed Galectin-1, programmed death-ligand 1 (PD-L1) and membrane-bound TGF-β1 that are key molecules involved in immunological tolerance. Some other publications also demonstrated immunoregulatory effects of MSC-EVs on T cell populations. Inhibition of activated T cell proliferation and generation of tolerogenic Treg cells were reported when activated splenocytes were cultured with EVs isolated from human ES-derived MSCs [63]. Blasquez et al. demonstrated inhibition of activation and differentiation of T cells cultured with MSC-EVs, which was associated with decreased IFN-γ release by CD4^+^ T cells [64]. One study reported that MSC-EVs exerted an immunosuppressive effect that was inferior to MSCs [65]. Recently, a dose-dependent reduction of T cell proliferation by EVs from UC-MSCs was also reported [66]. However, some discrepancies were reported in the literature. In 2015, Andrade et al. showed that MSC-EVs failed to suppress lymphocyte proliferation [67]. Other studies reported that MSC-EVs had no effect on proliferation and IFN-γ production by in vitro-stimulated primary T cells or sorted activated T cells [68,69]. Of interest, opposite effects of MSC-EVs and MSCs were reported. MSC-EVs did not affect proliferation of CD3^+^ T cells, but induced apoptosis of CD3^+^ and CD4^+^ T cells, and increased proliferation and apoptosis of Treg cells [70]. This was the first study discussing that both MSCs and MSC-EVs could exert immunomodulatory effects but with likely different underlying mechanisms.

Immunomodulatory effect of MSC-EVs has also been investigated on B lymphocytes. Inhibition of B cell proliferation and differentiation with a reduced secretion of immunoglobulins has been described [71]. A comparable approach reported that MSCs and MSC-EVs exhibited similar inhibitory activity on synthetic CpG-containing oligonucleotides (CpG)-induced B cell proliferation but MSCs were more effective than EVs to inhibit plasmacell differentiation and antibody secretion [65]. More recently, another group demonstrated that MSC-EVs inhibited B cell proliferation and also inhibited NK cells proliferation [69].

Finally, suppressive function of MSC-EVs on monocytes and macrophages was described. In 2017, Monguio-Tortajada et al. explored the effect of MSC-EVs on monocyte polarization using different stimuli. Cultured monocytes with MSC-EVs retained a non-activated state with no change in the expression of CD80, CD163, CD206 and IL-10 [66]. Indeed opposite to MSCs, MSC-EVs did not induce polarization towards M2 macrophages. By contrast, Lo Sicco et al. showed that exosomes were internalized by macrophages and elicited a switch from M1 to M2 phenotype [72]. Altogether, the results suggested that immunosuppressive properties of MSCs were at least in part mediated by release of EVs that conveyed regulatory molecules. Understanding the underlying mechanisms of MSC-EV functions will need however further investigation since MSC-EVs do not fully reproduce effects of parental MSCs.

### 4.2. Regenerative Properties of Extracellular Vesicles Derived from Mesenchymal Stem Cells

Regenerative activity, encompassing pro-angiogenic, anti-apoptotic, anti-fibrotic, proliferative activities of MSC-EVs have been largely investigated in several animal models including myocardial infarction, brain, lung, liver, and acute kidney injury [73]. Regarding pro-angiogenic activity of MSC-EVs, a recent study demonstrated protection against ischemic/reperfusion injury-induced kidney injury through pro-angiogenic effects [74]. Delivery of pro-angiogenesis related VEGF and RNAs were shown to be involved in this process. A proteomic analysis based on high-resolution isoelectric focusing coupled liquid chromatography tandem mass spectrometry revealed that MSC-EVs contained growth factors such as epithelial growth factor (EGF), FGF, platelet-derived growth factor (PDGF), and notably NFκB signaling pathway which have all been shown to contribute to pro-angiogenic activity [75]. Pro-angiogenic factors in EVs were released not only as soluble cytokines, but also as mRNA. Combination of proteic factor interacting with target endothelial cell membrane and delayed mRNA transferred via fusion inside the cells resulted in blood vessel induction with development of capillary-like structures [76].

Proliferative effects of MSC-EVs have been investigated in diverse models, notably in skin repair. In cutaneous wound healing, MSC-EVs could be taken up and internalized by fibroblasts to stimulate cell migration, proliferation and collagen synthesis [77,78,79]. Carbon tetrachloride (CCl4)-induced liver injury was attenuated by treatment with MSC-EVs, which induced increase in hepatocyte proliferation, as demonstrated with proliferating cell nuclear antigen (PCNA) elevation [80]. In a model of acute myocardial infarction in rats, cardiac function was improved in the animals treated with MSC-EVs [81]. This was attributed to accelerated endothelial cell proliferation and migration, tube-like structure formation in vitro, and blood vessel formation in vivo. In many cases, proliferative and anti-apoptotic activities of MSC-EVs were reported.

Anti-apoptotic effects of MSC-EVs have been evaluated in different models. For example, osteosarcoma cells were significantly resistant to apoptosis when exposed to MSC-EVs, which transported metastasis-associated genes, including monocarboxylate transporters, bone morphogenetic protein (BMP)-2, FGF-7, MMP-1, and focal adhesion kinase (FAK)-1 [82]. In another in vitro model of cisplatin-induced apoptosis of human renal tubular epithelial cells, analysis of MSC-EVs cargo revealed that anti-apoptotic genes, such as B-cell lymphoma extra-large (BCL-xL), BCL2 and Baculoviral IAP Repeat Containing 8 (BIRC8) were up-regulated and genes that have a central role in cell apoptosis such as caspase-1, caspase-8 and lymphotoxin alpha (LTA) were down-regulated [83]. In colitis, MSC-EVs suppressed apoptosis via reducing the cleavage of caspase-3, caspase-8 and caspase-9 in rats [84]. In renal injury of unilateral ureteral obstruction, MSC-EVs played an important anti-apoptotic role through modulation of miRNA profiles including miR-299, miR-499, miR-302, and miRNA-200 [85]. MSC-EVs were found to exert a pro-survival effect on renal cells in vitro and in vivo, by down-regulating apoptotic signals. 

Finally, anti-fibrotic effect of MSC-EVs has been evaluated in a number of studies. Implantation of MSC-EVs reduced surface fibrous capsules, alleviated hepatic inflammation and collagen deposition in CCl4-induced fibrotic liver [86]. MSC-EVs reduced expression of type I and III collagens and TGF-β1 and reduced Smad2 phosphorylation in liver. Similarly, in glycerol- or cisplatin-induced kidney injury, MSC-EVs reduced fibrosis and allowed recovery of renal function [87,88]. In these models, MSC-EVs ameliorated oxidative stress and reduced apoptosis resulting in kidney cell proliferation. 

### 4.3. Therapeutic Role of Extracellular Vesicles Derived from Mesenchymal Stem Cells in Rheumatic Diseases 

The large body of evidence that MSC-EVs exert similar pro-regenerative, anti-apoptotic, anti-fibrotic and anti-inflammatory functions as the parental cells provides new perspectives for their use in the treatment of OA and RA. To date, the role of MSC-EVs has not been documented in RA disease. Our recent data, however, indicated that MSC-EVs, namely exosomes and microparticles, can reduce arthritis signs in the CIA model. Therapeutic effect was related to the decrease in plasmablast population and increase in Breg cells secreting IL-10 [89].

In a model of osteochondral defect in immunocompetent rats, Zhang et al. demonstrated that MSC-EVs promoted cartilage regeneration [90]. In this study, injection of EVs accelerated neotissue filling and enhanced matrix synthesis of type II collagen and sulphated glycosaminoglycan (s-GAG). By 12 weeks, treated rats displayed complete restoration of cartilage and subchondral bone. Although this study did not report data in a model of OA, it demonstrated the efficacy of MSC-EVs in promoting cartilage repair in vivo.

In OA, two recent papers showed a therapeutic role of MSC-EVs. A beneficial effect of engineered MSC-EVs was reported in a rat model of knee OA [91]. In vitro, MSC-EVs promoted chondrocyte proliferation and migration but inhibited the secretion of ECM components. However, EVs from MSCs overexpressing miR-140-5p induced proliferation and migration of chondrocytes without decreasing ECM production. In vivo, increase of cartilage regeneration and prevention against the development of OA was demonstrated in rats treated with miR-140-5p-transporting MSC-EVs. In the second study, EVs isolated from synovium- or induced pluripotent stem cells (iPS)-derived MSCs were efficient to attenuate OA score in the CIOA model [92]. Our unpublished results demonstrated in vitro the capacity of MSC-EVs to reduce the expression of catabolic and inflammatory markers in IL1β-induced OA murine chondrocytes. In this model, we nicely demonstrated protection against cartilage and bone degradation using histomorphometric analysis of bone parameters by micro-computed tomography (µCT) and of cartilage parameters by confocal laser scanning microscopy analysis [93]. Therapeutic efficacy of MSC-EVs still needs to be demonstrated in larger animal models but first results in small animals are very encouraging.

## 5. Conclusions

Crosstalk between cells of the joint compartment through EVs is likely an important communication pathway. Understanding the messenger role of EVs in these communication pathways in tissue homeostasis and pathophysiological conditions is still at its beginning. In addition, exact contribution of different types of EVs, namely exosomes or microparticles, is far from being known and factors acting on their synthesis and release are not yet identified. However, there is no doubt that EVs are important mediators in the physiopathology of rheumatic diseases and the possibility of using them as biomarkers of these diseases is under investigation. This will require an improved classification system and a better standardization of the techniques used for purification and characterization. In parallel, usefulness of EVs isolated from different cells for therapeutic purposes is being evaluated in a variety of preclinical models. Much evidence of efficacy of EVs derived from MSCs has been reported in many animal models but is still poor for the treatment of rheumatic diseases. Future work on MSC-EVs is needed to decipher the mechanisms responsible for their therapeutic effect but the growing body of research on MSC-EVs will likely contribute to better define therapeutic options. This approach might represent one of the most promising and innovative strategies for RA and OA therapies.
